# Allocation of Heme Is Differentially Regulated by Ferrochelatase Isoforms in *Arabidopsis* Cells

**DOI:** 10.3389/fpls.2016.01326

**Published:** 2016-08-31

**Authors:** Nino A. Espinas, Koichi Kobayashi, Yasushi Sato, Nobuyoshi Mochizuki, Kaori Takahashi, Ryouichi Tanaka, Tatsuru Masuda

**Affiliations:** ^1^Graduate School of Science, The University of TokyoTokyo, Japan; ^2^Graduate School of Arts and Sciences, The University of TokyoTokyo, Japan; ^3^Graduate School of Science and Engineering, Ehime UniversityEhime, Japan; ^4^Graduate School of Science, Kyoto UniversityKyoto, Japan; ^5^Institute of Low Temperature Science, Hokkaido UniversitySapporo, Japan

**Keywords:** ferrochelatase, heme allocation, cytochromes, photosynthesis, plastid, biotic stress

## Abstract

Heme is involved in various biological processes as a cofactor of hemoproteins located in various organelles. In plant cells, heme is synthesized by two isoforms of plastid-localized ferrochelatase, FC1 and FC2. In this study, by characterizing *Arabidopsis* T-DNA insertional mutants, we showed that the allocation of heme is differentially regulated by ferrochelatase isoforms in plant cells. Analyses of weak (*fc1-1*) and null (*fc1-2*) mutants suggest that FC1-producing heme is required for initial growth of seedling development. In contrast, weak (*fc2-1*) and null (*fc2-2*) mutants of *FC2* showed pale green leaves and retarded growth, indicating that FC2-producing heme is necessary for chloroplast development. During the initial growth stage, *FC2* deficiency caused reduction of plastid cytochromes. In addition, although *FC2* deficiency marginally affected the assembly of photosynthetic reaction center complexes, it caused relatively larger but insufficient light-harvesting antenna to reaction centers, resulting in lower efficiency of photosynthesis. In the later vegetative growth, however, *fc2-2* recovered photosynthetic growth, showing that FC1-producing heme may complement the *FC2* deficiency. On the other hand, reduced level of cytochromes in microsomal fraction was discovered in *fc1-1*, suggesting that FC1-producing heme is mainly allocated to extraplastidic organelles. Furthermore, the expression of *FC1* is induced by the treatment of an elicitor flg22 while that of *FC2* was reduced, and *fc1-1* abolished the flg22-dependent induction of *FC1* expression and peroxidase activity. Consequently, our results clarified that FC2 produces heme for the photosynthetic machinery in the chloroplast, while FC1 is the housekeeping enzyme providing heme cofactor to the entire cell. In addition, FC1 can partly complement FC2 deficiency and is also involved in defense against stressful conditions.

## Introduction

Heme (protoheme) is a cofactor responsible for various biological processes including oxygen metabolism, oxygen transfer, electron transfer, and secondary metabolism. Heme also functions as a regulatory molecule (Tanaka et al., 2011).

In plants, heme biosynthesis takes place in plastids by sharing the pathway with chlorophyll biosynthesis until formation of protoporphyrin IX (Masuda et al., 2003; Tanaka et al., 2011). Heme is formed directly from protoporphyrin IX by insertion of ferrous (Fe^2+^) ion, whereas chlorophylls are synthesized by several steps after insertion of magnesium ion into protoporphyrin IX. The insertion of Fe^2+^ into protoporphyrin IX is catalyzed by ferrochelatase. In land plants, two ferrochelatase isoforms of cyanobacterial origin have been identified (Chow et al., 1998; Suzuki et al., 2002), ferrochelatase 1 (FC1) and ferrochelatase 2 (FC2), whose amino acid sequences are about 69% identical (Chow et al., 1998). A characteristic feature of FC2 is its hydrophobic C-terminal extension, which includes a putative light harvesting chlorophyll *a*/*b*-binding (LHC) motif (also called CAB domain) (Suzuki et al., 2002). This conserved LHC motif is also present in cyanobacterial ferrochelatase and in FC2-type ferrochelatases from other higher plants. In cyanobacterium *Synechocystis* sp. PCC 6803, it is reported that the LHC motif is not required for catalytic activity but is essential for dimerization of the ferrochelatase (Sobotka et al., 2010).

These two ferrochelatase isoforms show a clear contrast in gene expression profile such that *FC2* is mainly expressed in photosynthetic tissues, whereas FC1 is expressed in all tissues (Chow et al., 1998; Suzuki et al., 2002). Particularly in roots, the *FC1* expression is predominant and the *FC2* expression is hardly detected, suggesting that FC1 and FC2 have different roles among various tissues. Furthermore, FC1 is strongly upregulated by wounding and oxidative stresses in photosynthetic tissues (Singh et al., 2002; Nagai et al., 2007). Since *FC1* is co-induced with genes encoding endoplasmic reticulum (ER)-localized cytochrome P450 family and cytosolic ascorbate peroxidase upon wounding, it is presumed that FC1 supplies extraplastidic heme for defensive functions (Nagai et al., 2007). Actually, Genevestigator analysis showed stress-responsive induction of *FC1*, in response to drought, wounding, and reactive oxygen species, but not *FC2* (Scharfenberg et al., 2015). Meanwhile, FC2 is proposed to be involved in heme production for photosynthetic cytochromes. In fact, gene ontology analysis revealed that genes associated with the term ‘photosynthesis’ are significantly enriched in the co-expressed genes with *FC2*. It should be noted that co-expression analysis by Scharfenberg et al. (2015) suggested that genes associated with the term ‘defense response to bacteria,’ which includes reactions triggered by the pathogens such as *Pseudomonas syringae*, was identified as other enriched categories of *FC2* co-expressed genes.

Mutants of ferrochelatase isoforms have so far been characterized. For FC1, a knock-down mutant (*fc1-1*) was characterized by Nagai et al. (2007) showing reduced heme levels particularly in roots, but photosynthetic parameters such as chlorophyll and carotenoid content, and the efficiency of photosystem II (PSII), were essentially unaffected (Nagai et al., 2007). Woodson et al. (2011) reported that a homozygous null mutant (*fc1-2*) of *FC1* could not be recovered from heterozygous parents, suggesting an embryonic-lethal phenotype. Further analysis of this mutant suggests that a second (unlinked) T-DNA insertion may be present that could also cause the lethal phenotype (Scharfenberg et al., 2015). For FC2, weak (*fc2-1*) and null (*fc2-2*) T-DNA insertion mutants have been isolated (Scharfenberg et al., 2015; Woodson et al., 2015). Phenotypic analysis of *fc2-1* showed that the mutant seedlings are abnormally small with pale green rosette leaves, low in chlorophylls, carotenoids and several photosynthetic proteins, and impaired photosynthetic performance (Scharfenberg et al., 2015; Woodson et al., 2015). Moreover, it was found that the lack of FC2 resulted in a *fluorescent* (*flu*)-like phenotype (Scharfenberg et al., 2015; Woodson et al., 2015). In the *flu* mutant, the photosensitizer protochlorophyllide accumulates in the dark (Meskauskiene et al., 2001). Consequently, exposure of the *flu* mutant to light generates singlet oxygen (^1^O_2_) and *flu* seedlings bleach and die. Although accumulating species of tetrapyrroles are different between Scharfenberg et al. (2015) (i.e., protochlorophyllide accumulation) and Woodson et al. (2015) (i.e., protoporphyrin IX accumulation), *fc2-1* and *fc2-2* were found to exhibit *flu*-like phenotype when they are grown in the dark unlike *fc1-1* mutant.

In addition to the differences in gene expression, a distinct involvement of FC1- and FC2-derived heme in retrograde plastid signaling has been proposed (Woodson et al., 2011). Woodson et al. (2011) performed a gain-of-function genetic screening of *g*enomes*-un*coupled (*gun*) mutants using activation-tagged lines of *Arabidopsis*, and subsequently discovered that overexpression of *FC1* restores nuclear-encoded photosynthesis-associated gene expression even when chloroplast development is blocked. These data suggest that increased flux through the FC1-producing heme may act as a signaling molecule that control photosynthesis-associated nuclear genes as retrograde signal. Although FC1 and FC2 colocalized to the same plastids and utilized the same biosynthetic pathway, overexpression of *FC2* failed to derepress photosynthesis gene expression (Woodson et al., 2011). Furthermore, genetic complementation of *fc2-1* showed that expression of FC1 could not prevent the accumulation of protoporphyrin IX, but restored wild-type levels of heme and chlorophyll in constant light and protochlorophyllide in the dark (Woodson et al., 2015). These results suggest that although FC1 and FC2 are colocalized in plastids and function for heme biosynthesis, FC2-derived heme is allocated differently from FC1-derived heme that can be transferred to extraplastidic locations and function in stress-responses or retrograde signaling. However, the allocation of heme produced by each ferrochelatase isoforms in plant cells is not well understood.

In this study, we re-examined T-DNA insertional *Arabidopsis* mutants deficient in ferrochelatase isoforms. By further analysis of these mutants, we showed that FC1 and FC2 have distinct physiological functions for developmental growth. Furthermore, these isoforms are distinctly involved in heme allocation inside and outside plastids. Thus, our data demonstrate that the allocation of heme is differentially regulated by FC1 and FC2 in plant cells.

## Materials and Methods

### Plant Materials and Growth Conditions

The *Arabidopsis* T-DNA insertional mutants of ferrochelatase isoforms, *fc1-1* (SALK_150001), *fc1-2* (GK_110D_02), *fc2-1* (GK_766_H08), and *fc2-2* (SAIL_20_C06), are Columbia ecotype and obtained from ABRC stock center. Seeds were surface-sterilized before sowing on solidified Murashige and Skoog medium (Murashige and Skoog, 1962) containing 1% (w/v) sucrose and 1% (w/v) gelrite (Duchefa) at 22°C under continuous white light (35–45 μmol photons m^-2^ s^-1^). All data represent three biological replicate experiments. For flg22 (Sawady Technology, GenScript) treatment, sterilized seeds were cultured in 15 ml of 0.1x MS liquid medium containing 0.1% sucrose (w/v) and buffered to pH 5.7 with 0.05 g l^-1^ MES at 110 rpm, 22°C, 60 μmol photons m^-2^ s^-1^ on a gyratory shaker (Shake-LR, Taitec) for indicated days, added indicated concentration of flg22, and cultured for indicated periods.

### Genotyping and Quantitative Reverse Transcriptase-Polymerase Chain Reaction (qRT-PCR) Analysis

Genomic DNA and total RNA were isolated using Nucleon Phytopure Plant Extraction Kit (Thermo Scientific) and RNeasy Mini Kit (Qiagen), respectively, and nucleotide concentration was determined using NanoDrop (ND-1000; Thermo Scientific) spectrophotometer. For genotyping, isolated DNA was subjected to PCR amplification with specific primers and obtained products were electrophoresed in 1% agarose gel. For qRT-PCR, 1 μg of total RNA was reverse transcribed using PrimeScript II 1st strand cDNA Synthesis Kit (Takara). qRT-PCR was performed with specific primer sets using Thunderbird SYBR qPCR Mix (Toyobo) on a Mini Opticon Real-Time PCR System (Bio-Rad). *ACTIN8* was used to compute the relative transcript abundance. All primers used are listed in Supplementary Table 1.

### Measurement of Photosynthetic Pigments

Chlorophyll content was measured as described (Arnon, 1949; Melis et al., 1987). Heme contents were measured by highly sensitive assay using horseradish peroxidase as described (Masuda and Takahashi, 2006; Takahashi and Masuda, 2009; Espinas et al., 2012).

### GUS Staining

By using *FC1* promoter fused β-glucuronidase (*GUS*) transgenic line (*FC1pro::GUS*), histochemical analyses for *GUS* expression were carried out as described (Nagai et al., 2007).

### Protein Extraction and Western Blot Analysis

Total and separated proteins were subjected to SDS-PAGE. Soluble (S) and membrane fractions (M) were prepared by centrifugation of plant homogenates for 10 min at 10,000 *g* under 4°C. Protein contents were determined using *RC DC* Protein Assay (Bio-Rad). In each lane, 40 μg of proteins were loaded. After SDS-PAGE in 12.5% polyacrylamide gels, proteins were electrophoretically transferred to a nitrocellulose membrane (Hybond-N^+^, Thermo Scientific) and subsequently exposed to antibodies tested. The blot was then incubated with anti-rabbit immunoglobulin G conjugated to horseradish peroxidase, following which the proteins were detected using chemiluminescence reagent (Millipore). All antibodies used are listed in Supplementary Table 2.

### Blue Native PAGE

For Blue Native PAGE, thylakoid membranes were isolated from the leaves of 2-week-old plants as described (Takahashi et al., 2014). Solubilized membrane proteins containing 5 μg chlorophyll were then separated by 5–14% acrylamide gradient gels according to the method of Wittig et al. (2006).

### Chlorophyll Fluorescence Measurement

Photochemical efficiency analysis was performed using a pulse amplitude modification (PAM) fluorometer (Junior-PAM, Walz) from the leaves of 2-week-old plants as described (Kobayashi et al., 2013). From the obtained fluorescence yields (*F*_o_, *F*_m_, F′_o_, and F′_m_), photosynthetic parameters were calculated according to the previous equations (van Kooten and Snel, 1990; Maxwell and Johnson, 2000). The Φ_NPQ_ and Φ_NO_ were determined according to the method of Kramer et al. (2004).

### Heme Staining

Microsomal light membrane (LM) fraction was extracted by layering membrane fractions on 0.81 M sucrose, followed by ultracentrifugation (Optima, Beckman, Inc.) for 1 h at 100,000 *g*. This fraction was solubilized in 62.5 mM Tris-HCl, pH 6.8, 4% SDS, 20% glycerol, and 0.2% bromothymol blue. After separation by non-reducing SDS-PAGE, gels were stained with 6.3 mM 3,3′,5,5′-tetramethylbenzidine (Nacalai Tesque) in methanol and 0.25 M sodium acetate in 3:7 (v/v) ratio, respectively. Hydrogen peroxide (H_2_O_2_) was then added to a final concentration of 30 mM. The visible staining was scanned using a flatbed scanner.

### Peroxidase Assay

Plants were grown on soil for 27 days as described (Nozaki et al., 2012). Excised rosette leaves were soaked and vacuum infiltrated with a solution of 0.5 μg ml^-1^ flg22 containing 0.001% Triton X-100, incubated in the solution for 24 h at 22°C in darkness and stored at -60°C. Extraction of peroxidase activities was carried out as described (Sato et al., 2011). Briefly, the stored leaves were homogenized in 50 mM Tris-HC1 buffer (pH 7.2) using a plastic pestle and then using an ultrasonic disruptor (UD-200, Tomy Seiko). The homogenate was centrifuged at 1,500 *g* for 5 min. The supernatant was re-centrifuged at 15,000 *g* for 20 min and used for assay of soluble peroxidase activities. Ionically bound peroxidase activities were extracted with 1 M NaCl, 50 mM Tris-HCl buffer (pH 7.2) from the pellet of cell walls. Peroxidase activities were measured as described (Sato et al., 1993). The final reaction mixture (100 μl) contained 5 μl of enzyme preparation, 13 mM guaiacol, 15 mM CaC1_2_, 5 mM H_2_O_2_, and 40 mM Tris-HCl (pH 7.2). The sum of soluble and ionically bound peroxidase activities was regarded as total peroxidase activity.

### Lignin Assay

For disruption, 5–6 seedlings frozen in liquid nitrogen were disrupted with a bead-type cell disrupter (MS-100, Tomy Seiko). Lignin assay was carried out as described (Schenke et al., 2011). For conversion to relative units based on absorbance the following relation was used: 100 μg lignin in 1 ml produce an *A*_280_ of 0.60 in a 1-cm cell according to Müsel et al. (1997).

### Statistics

All statistics were performed using two-tailed Student’s *t*-test. Asterisk indicate significant difference in *P* < 0.05 to wild-type control.

## Results

### Analysis of Mutants of Ferrochelatase Isoforms

In this study, to analyze physiological functions of ferrochelatase isoforms, we firstly re-examined the T-DNA insertion mutants of ferrochelatase isoforms in *Arabidopsis thaliana*.

**FIGURE 1:**
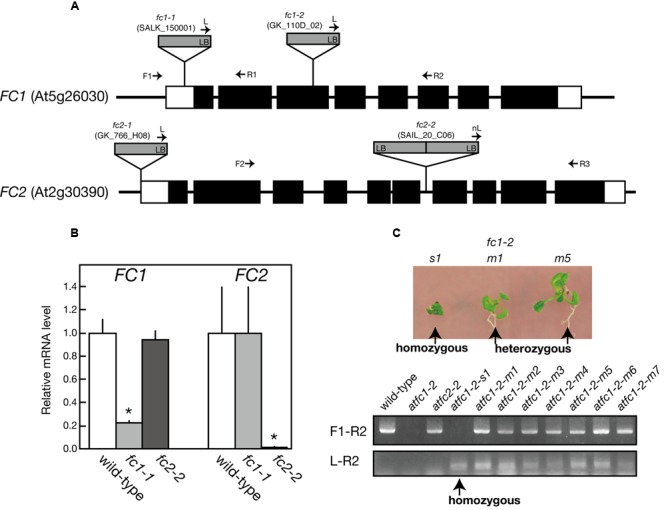
**Characterization of T-DNA insertional mutants of ferrochelatase isoforms. (A)** Schematic representation and T-DNA tagging of *FC1* (At5g26030) and *FC2* (At2g30390) loci. Exons (black boxes) and untranslated regions (white boxes) are shown. Location and orientation of T-DNA insertions in each line are indicated. Arrows represent the primers used for genotyping of *FC1* and *FC2* (see Supplementary Table 1). **(B)** qRT-PCR analysis of *FC1* and *FC2* mRNA transcripts extracted from 7-day-old wild type, *fc1-1*, and *fc2-2* seedlings. Values are presented as the fold difference from the wild-type after normalizing to the control gene *ACTIN8*. Bars indicate standard error of the mean (SEM) from three independent experiments. Asterisks indicate a significant difference from the wild-type (^∗^*P* < 0.05, Student *t*-test). **(C)** Phenotypic and genomic analysis of *fc1-2*. (Upper) Photographs of small (*s1*) and middle (*m1* and *m5*) size seedlings germinated from heterozygous *fc1-2* seeds. (Lower) PCR-based genomic analysis of these seedlings, showing small size seedling (*s1*) is actually homozygous *fc1-2* seedling.

**FIGURE 2:**
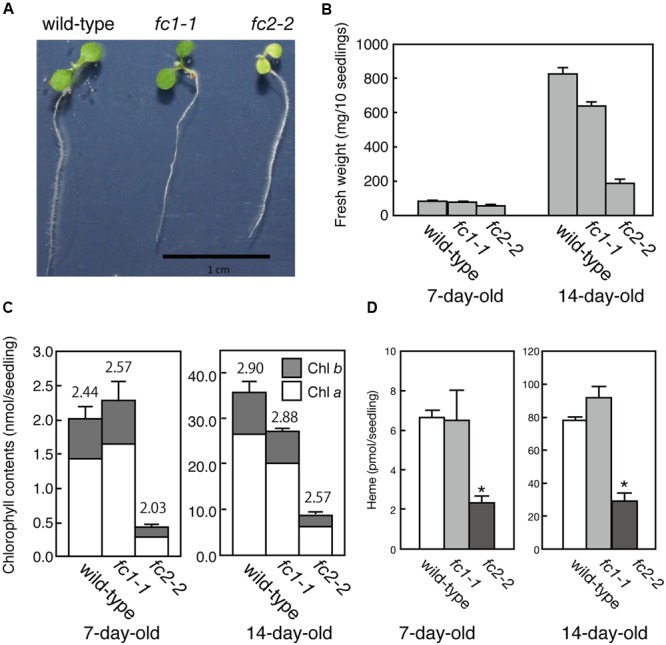
**Phenotypic characterization of *fc1-1* and *fc2-2*. (A)** Photographs of 7-day-old wild-type, homozygous *fc1-1*, and homozygous *fc2-2* seedlings. Seedlings of *fc2-2* show pale-green phenotype. **(B)** Fresh weights of wild-type, *fc1-1*, and *fc2-2* of 7- and 14-day-old seedlings. **(C)** Chlorophyll contents of wild-type, *fc1-1*, and *fc2-2* of 7- and 14-day-old seedlings. Chlorophyll *a* (white bars) and *b* (gray bars) contents are indicated. Numbers on the bars are chlorophyll *a*/*b* ratio. **(D)** Total endogenous heme contents in 7- and 14-day-old seedlings in wild-type, *fc1-1*, and *fc2-2.* Asterisks indicate a significant difference from the wild-type (^∗^*P* < 0.05, Student *t*-test). In all figures, bars indicate standard error of the mean (SEM) from three independent experiments.

For FC1, *fc1-1* (SALK_150001) and *fc1-2* (GK_110D_02) have so far been characterized (Nagai et al., 2007; Scharfenberg et al., 2015; Woodson et al., 2015). *fc1-1* possesses a single copy of T-DNA in the 5′-untranslational region of *FC1*, while in *fc1-2*, T-DNA insertion was localized in the third exon of *FC1* (**Figure 1A**). As reported previously (Nagai et al., 2007), *fc1-1* was almost comparable to wild-type phenotype (**Figure 2**; **Supplementary Figure S1**) and lacked stress-induced *FC1* expression (**Figure 8A**). qRT-PCR analysis showed about 80% reduction in the transcript levels of *FC1* in *fc1-1* even under normal growth condition (**Figure 1B**). Meanwhile, it was reported that a homozygous null *fc1-2* mutant is likely to be embryonic lethal (Woodson et al., 2011). Subsequently, it was suggested that the presence of a second unlinked T-DNA insertion is involved in the lethal phenotype of this line (Scharfenberg et al., 2015). Here, we found that some of homozygous *fc1-2* seeds were able to germinate, emerging as a very small seedling (**Figure 1C**). The germination rate was quite low (<1%), which is far lower than theoretical segregation rate of homozygous *fc1-2* (25%). PCR-based genome analysis confirmed that the small seedlings are actually homozygous *fc1-2* mutant (**Figure 1C** lower panel). The homozygous *fc1-2* was green but its growth was severely retarded and it died during initial seedling stage, suggesting that homozygous *fc1-2* can be occasionally successful for embryogenesis but seedlings are arrested during further development. Because *fc1-2* seedling was too small and its occurrence was too rare to be assayed, it was excluded from further analysis.

For *FC2, fc2-1* (GK_766_H08) and *fc2-2* (SAIL_20_C06) have so far been characterized (Scharfenberg et al., 2015; Woodson et al., 2015). In *fc2-1*, T-DNA insertion is located in the 5′-untranslated region, while in *fc2-2*, a single copy of T-DNA in tandem is inserted between exons 6 and 7 of *FC2* (**Figure 1A**). Here we analyzed *fc2-2* that shows more severe phenotype than *fc2-1* (Scharfenberg et al., 2015). After obtaining homozygous *fc2-2* mutant, we observed the deficiency of the *FC2* mRNA (**Figure 1B**) in the mutant. Consistent with previous reports (Scharfenberg et al., 2015; Woodson et al., 2015), *fc2-2* showed pale-green phenotype with substantial decrease in their weight and chlorophyll content during 1 week after germination (**Figures 2A–C**). In *fc2-2*, the chlorophyll *a*/*b* ratio was lower than that of wild-type, suggesting relatively high abundance of LHC proteins to reaction centers (**Figure 2C**). During further development for 1 more week, *fc2-2* became greener (**Figures 2B,C**) and finally it behaved like wild-type and became fertile (**Supplementary Figure S1D**). The levels of total heme per seedling in *fc2-2*, however, remained almost 30% of wild-type during 2 weeks after germination (**Figure 2D**). We could not detect the reduction of heme in *fc1-1* probably because the effect of FC1 deficiency in heme production is rather limited in roots and the difference of heme was not detectable in the seedling level (Nagai et al., 2007). The level of reduction of total heme in *fc2-2* were similar to that reported by Woodson et al. (2015), but more severe than those observed by Scharfenberg et al. (2015). It should be noted that consistent with previous studies (Scharfenberg et al., 2015; Woodson et al., 2015) *fc2-2* showed *flu* phenotype when they were grown in the dark (data not shown).

We also produced *fc1-1 fc2-2* double mutant by crossing (**Supplementary Figures S1A,B**). The obtained homozygous *fc1-1 fc2-2* double mutant showed more severe phenotype than parental lines with much reduced size and paler cotyledons than those of *fc2-2* (**Supplementary Figures S1B,C**). On MS solidified medium or soil, the homozygous *fc1-1 fc2-2* double mutant seedlings stopped their growth before or soon after bolting and died, so they are infertile (**Supplementary Figures S1D,E**). These results show that FC1 and FC2 have distinct physiological functions for developmental growth. Considering severe phenotype of *fc1-2, FC1* supplies heme essential for housekeeping function including embryogenesis. FC2-produced heme seems important for chloroplast development but not essential for seedling development. In the later developmental stage, it is likely that the deficiency of FC2 can be complemented by the function of FC1, but not vice versa.

### Histochemical Analysis of *FC1* Expression during Development

**FIGURE 3:**
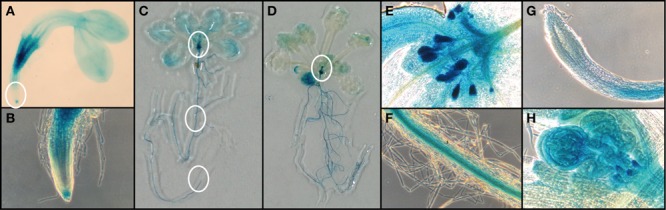
**Histochemical analysis of *FC1pro::GUS* line. (A)** *FC1*-dependent GUS activities in 2-day-old seedlings. **(B)** Zoomed image of regions of root cap and elongation zone as indicated by a white circle in **(A)**. Representative images of **(C)** 14-d-old seedling and **(D)** 21-day-old seedling. Zoomed image of regions of **(E)** primordial leaves, **(F)** root veins, and **(G)** veins as indicated by white circles in **(C)**. **(H)** Zoomed image of primordial bolting stem as indicated by a white circle in **(D)**.

To observe the expression of *FC1* during development, we performed histochemical analysis of *FC1* by using *FC1* promoter fused *GUS* line (*FC1pro::GUS*) (Nagai et al., 2007) (**Figure 3**). In young 2-day-old seedlings (**Figures 3A,B**), *FC1* expression was mainly observed in roots, with prominent staining in regions of vein and root cap, but not elongation zone. In cotyledons, faint staining was observed in vein regions. In mature seedlings (2- and 3-week-old), *FC1* expression was also mainly observed in roots (**Figures 3C,D**). High expression of *FC1* was detected in primordial tissues of leaves and stipules in 2-week-old seedlings and bolting shoot in 3-week-old seedlings (**Figures 3C–E,H**). Meanwhile, in developed leaves, faint expression of *FC1* was observed, and its expression was much reduced in 3-week-old seedlings when compared to 2-week-old seedlings (**Figures 3C,D**). In roots of matured plants, the expression of *FC1* was mainly observed in vein regions but not in root caps (**Figures 3F,G**). These results show that in addition to the housekeeping expression in roots, *FC1* is highly expressed in primordial newly emerging tissues, such as new leaves, stipules, and bolting stems, suggesting its important role for the activity of the apical shoot meristem (**Figures 3C–E,H**). Since *FC2* is preferentially expressed in photosynthetic tissues (Suzuki et al., 2002), and its expression is highly analogous to other photosynthetic genes (Scharfenberg et al., 2015), histochemical analysis of *FC2* expression was not performed in this study.

### Effect of FC2 Deficiency on the Assembly of Photosynthetic Machineries

**FIGURE 4:**
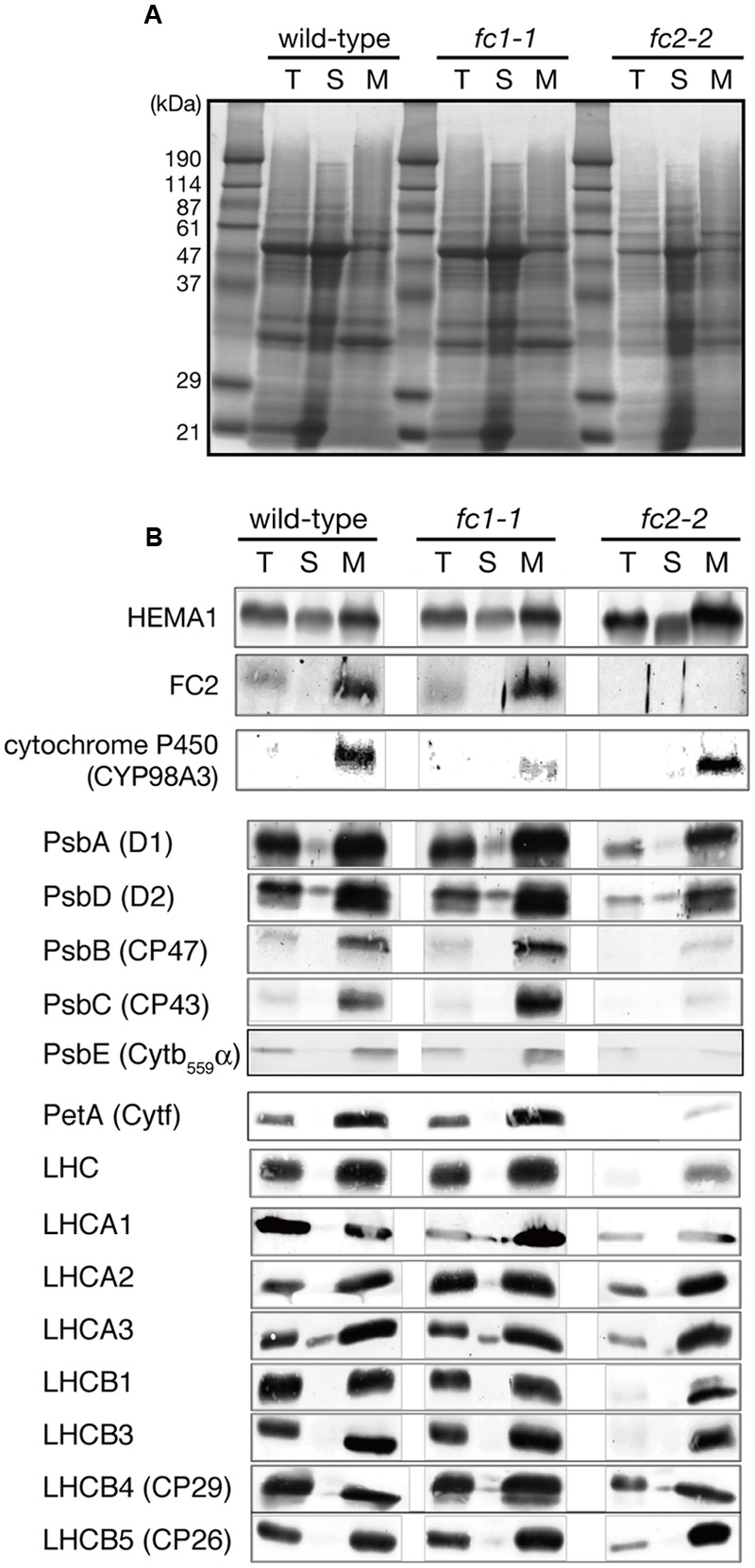
**Western blot analysis of heme-associated proteins and hemoproteins in ferrochelatase-deficient mutants. (A)** CBB staining of SDS-PAGE. Samples of total (T), soluble (S), and membrane (M) fractions (40 μg each) were loaded on the gel. **(B)** Western blot analysis of glutamyl-tRNA reductase (HEMA1), FC2, cytochrome proteins, core subunits of PSII complexes, and peripheral LHC proteins. Note that all cytochromes and FC2 were detected in membrane fraction, while HEMA1 was also detected in soluble fraction. “LHC” means polyclonal antibodies that recognize multiple LHC proteins were used for detection.

We then performed the immunoblot analysis on *fc1-1* and *fc2-2* mutants. Each sample was loaded on the same amount of protein basis. As shown in **Figure 4**, FC2 protein was undetectable in *fc2-2*, confirming null mutation of *FC2.* Cytochromes *b_6_f* is present in chloroplast and functions in the photosystem electron transport chain. As shown by immunoblot analysis in **Figure 4**, the amount of cytochrome *f* (PetA) was pronouncedly reduced in *fc2-2*, indicating that formation of cytochrome *b_6_f* in chloroplasts requires heme supply from FC2-dependent pathway. We noticed that the level of α subunit of cytochrome *b_559_* (PsbE) was also severely reduced in *fc2-2* (**Figure 4B**). Meanwhile, the levels of both plastid cytochromes were almost unaffected in *fc1-1*. These results suggest that heme allocated to chloroplasts is mainly attributed by FC2-dependent pathway. On the other hand, the contribution of FC1 to heme supply to chloroplasts may be limited and therefore FC1 cannot complement the chloroplast defects in *fc2-2* during the initial stage of development. We also found that the level of HEMA1, a predominant isoform of glutamyl-tRNA reductase, is certainly accumulated in *fc2-2* (**Figure 4B**). HEMA1 is a rate-limiting enzyme of total flow of tetrapyrrole biosynthesis (Tanaka et al., 2011), so the supply of porphyrin intermediates may be increased in *fc2-2*.

**FIGURE 5:**
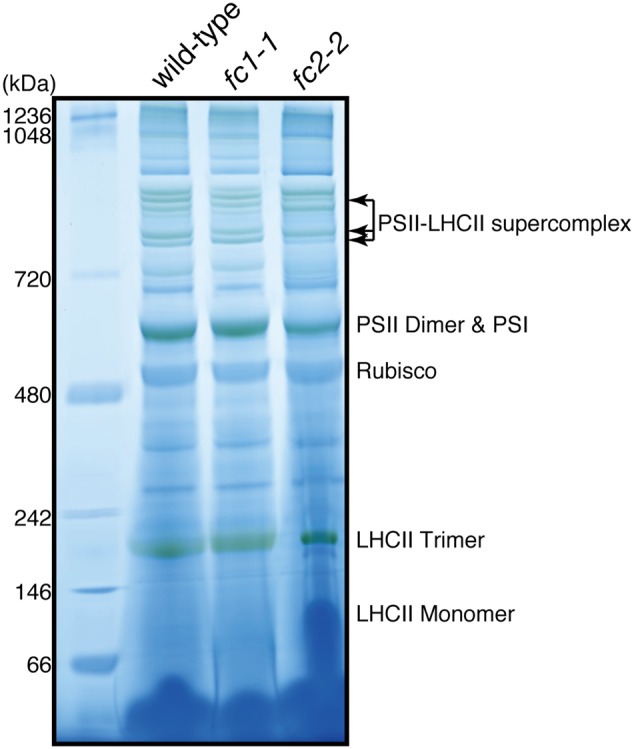
**Blue-native PAGE analysis of photosystem complexes in wild-type and ferrochelatase deficient mutants.** Thylakoid membrane fractions obtained from 14-day-old seedlings are solubilized. Proteins containing 5 μg chlorophyll were loaded to 4–14% linear gradient gel. Molecular size markers are indicated on the left.

We also determined the levels of photosynthetic proteins (**Figure 4B**). In *fc2-2*, similar to the reduction of cytochrome *b_6_f*, reduction of other photosynthetic proteins was observed. It seems that the FC2 deficiency globally affected the amounts of photosynthetic machinery, such as reaction center proteins as well as LHC antenna proteins. Although the levels of reduction were varied among proteins, the levels of reduction of photosynthetic proteins were less pronounced than those of cytochromes *f* and PsbE. Exceptions are CP43 and CP47 proteins showing much severe reduction than any other photosynthetic proteins. We then performed blue native gel analysis of membrane proteins loaded on the same amount of chlorophyll basis (**Figure 5**). When compared to wild-type and *fc1-1, *fc2-2** deficiency caused slightly different band profile of PSII-LHCII supercomplex, probably because of different composition of PSII subunits (**Figure 4B**). Comparing to reaction center complexes, relative band intensities of LHCII trimer and monomer bands were higher in *fc2-2*. Considering a lower chlorophyll *a*/*b* ratio, relatively higher amounts of LHC proteins to reaction center proteins may be present in *fc2-2.* It is likely that heme deficiency in *fc2-2* mainly affected the assembly and connection of LHC antenna to reaction center complexes, but not the assembly of PSI and PSII reaction center complexes.

**FIGURE 6:**
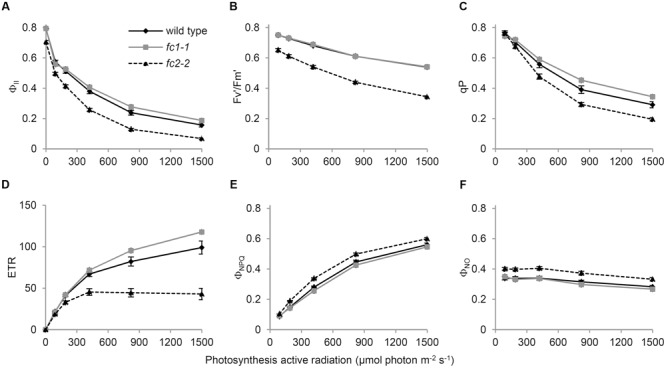
**Chlorophyll fluorescence parameters of wild-type and ferrochelatase deficient mutants.** Light response curves of **(A)** Φ_II_, **(B)** *F*_v_′/F_m_′, **(C)** qP, **(D)** ETR, **(E)** Φ_NPQ_, and **(F)** Φ_NO_ are shown. Φ_II_, PSII quantum yield/operating quantum efficiency of PSII photochemistry; *F*_v_′/F_m_′, efficiency of open PSII reaction centers; qP, fractions of PSII centers in open states based on puddle model for the photosynthetic unit; ETR, electron transfer rate in PSII; Φ_NPQ_, quantum yield of light-induced non-photochemical quenching (NPQ)/NPQ efficiency; Φ_NO_, non-regulated energy dissipation. PAR means photosynthetic active radiation. Bar indicates SEM from five independent experiments for wild type and *fc2-2*. In addition, two biological replicates of the *fc1-1* data were included as comparison.

To further examine the impact of FC1 or FC2 deficiency on the photosynthetic electron flow, we analyzed their chlorophyll fluorescence using PAM techniques (Maxwell and Johnson, 2000). The light-response curves of Chl fluorescence from PSII showed that the effective photochemical quantum yield of PSII (Φ_II_) in *fc2-2* was lower than those in wild-type and *fc1-1* under low to high photosynthetically active radiation (PAR) (**Figure 6A**). This trend is consistent with previous observation using *fc2-1* (Scharfenberg et al., 2015), but much more pronounced in *fc2-2*. In **Figure 6A**, the Φ_II_ values in darkness (0 μmol photon m^-2^ s^-1^ PAR) represent the maximum quantum yield of PSII (*F*_v_/*F*_m_), and that was decreased to 0.70 in *fc2-2* compared with 0.79 in wild type. Consistently, the maximum quantum yield of open (oxidized) PSII under light (F′_v_/F′_m_) was lower in *fc2-2* than the wild type and *fc1-1* under all ranges of light intensity (**Figure 6B**). These data suggest that the intrinsic photochemical activity of PSII is impaired in *fc2-2*. Moreover, the fraction of open PSII represented by coefficient of the photochemical quenching (qP) was decreased in *fc2-2* compared to wild-type and *fc1-1* (**Figure 6C**). Electron transport rate (ETR) of PSII calculated from Φ_II_ was substantially lower in *fc2-2* with reaching to a plateau at lower PAR than the wild type and *fc1-1* (**Figure 6D**). Thus, decreased openness of PSII with decreased intrinsic photochemical efficiency resulted in retarded photosynthetic electron transport in *fc2-2*.

Absorbed light energy by LHCII-PSII can be divided into Φ_II_, quantum yield of light-induced non-photochemical quenching (Φ_NPQ_) and quantum yield of non-regulated energy dissipation (Φ_NO_) (Kramer et al., 2004). In *fc2-2*, decreased Φ_II_ was reflected in higher values of both Φ_NPQ_ (**Figure 6E**) and Φ_NO_ (**Figure 6F**). These results suggest that in *fc2-2*, the absorbed light energy is not efficiently transferred from LHC antenna to PSII reaction centers and dissipated as heat or fluorescence.

### Impact of FC1 Or FC2 Deficiency on Hemoproteins

Our previous study showed that *FC1* was co-expressed with wounding-inducible cytochrome P450 family (Nagai et al., 2007), implying a relationship between FC1-dependent heme pathway and cytochrome P450 proteins. To confirm whether the level of cytochrome P450 is affected in *fc1-1* or not, we examined the abundance of a representative cytochrome P450 (CYP98A3) by immunoblot analysis (**Figure 4B**). CYP98A3, whose mRNA can be detected in all organs of *Arabidopsis*, is involved in the conversion of *p*-coumaroyl quinate into chlorogenic acid, and also is expected to catalyze the *meta*-hydroxylation step for the formation of lignin monomers (Bak et al., 2011). The level of CYP98A3 was actually reduced in *fc1-1*, while it remained to the wild-type level in *fc2-2* (**Figure 4B**), showing that the FC1-dependent pathway and not FC2 is required for the accumulation of ER-localized CYP98A3.

**FIGURE 7:**
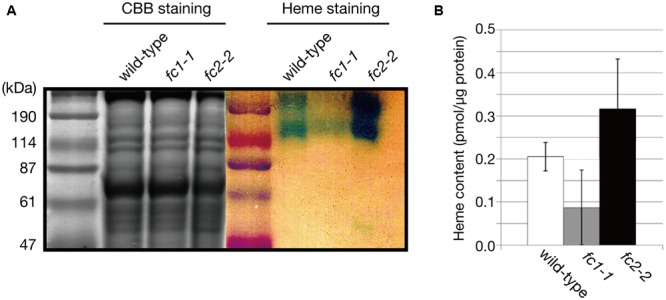
**Heme staining of ER-enriched microsomal light membrane (LM) fraction. (A)** CBB and heme staining of the LM fraction (30 μg/well) of wild type, *fc1-1*, and *fc2-2*. Blue staining represents the heme-dependent peroxidase activity. **(B)** Heme levels in LM fractions were determined by highly sensitive heme assay. Bars indicate SEM from three independent experiments.

The decrease in CYP98A3 in *fc1-1* suggests an essential role of FC1 in heme supply to cytochrome P450 proteins. Since almost all plant cytochrome P450 proteins are known to bind the cytoplasmic surface of ER (Bak et al., 2011), we isolated the ER-enriched microsomal LM fraction from seedlings and performed heme staining (**Figure 7A**). In this experiment, hemoproteins in non-reducing gel were detected by peroxidase activity of heme. As shown in **Figure 7A**, two major bands (220–240 and 120 kDa) were detected, which are presumably representing cytochrome P450 proteins as oligomeric forms. Interestingly, *fc1-1* showed less staining of these bands, while *fc2-2* showed more intense staining than wild-type. To test whether the levels of staining are actually related to the extractable heme levels, we performed heme determination in LM fraction with highly sensitive heme assay (Takahashi and Masuda, 2009; Espinas et al., 2012) (**Figure 7B**). Because of very low heme levels in each fraction, the obtained data were fluctuated. Although heme levels in each fraction showed trends similar to heme staining data, they were not statistically validated. Considering the total heme level was unchanged in *fc1-1* and decreased in *fc2-2*, heme produced by FC1 and FC2 may allocate differentially to various organelles. Our data suggest that FC1 is involved in supply of extraplastidic heme to LM fraction, while FC2 is not. Furthermore, under FC2 deficiency, such extraplastidic heme supply is likely to be upregulated. It is interesting to note that, in *fc2-2*, the abundance of the HEMA1 protein was certainly increased (**Figure 4B**) whereas the expression of *FC1* was not upregulated (**Figure 1B**). The accumulation of HEMA1 by the FC2 deficiency may increase the global flow of heme biosynthetic pathway and result in the increased heme supply to ER through the FC1 activity in *fc2-1*. Alternatively, in *fc2-2*, a decrease in heme supply inside chloroplasts may also lead to the activation of the HEMA1 enzyme because heme can function as a feedback regulator of HEMA1.

### FC1 is Involved in Flg22-Dependent Stress Responses

**FIGURE 8:**
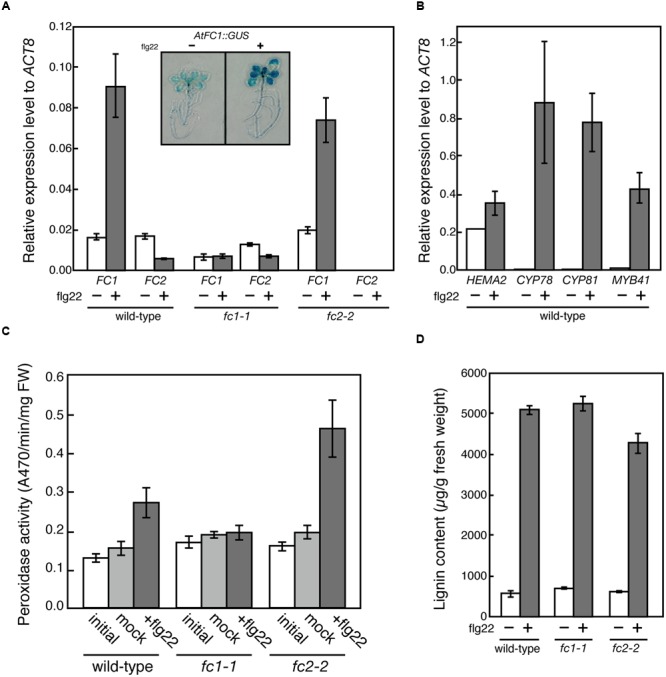
**flg22-dependent induction of FC1. (A)** qRT-PCR analysis of FC1 and FC2 in wild-type (WT), *fc1-1*, and *fc2-2* treated with 1 μg ml^-1^ flg22 for 6 h after 5 days of culture. Inset is photograph of GUS staining of *FC1::GUS* line treated with or without flg22. **(B)** qRT-PCR analysis of stress-responsive genes (*HEMA2, CYP78, CYP81*, and *MYB41*) in wild-type treated with 1 μg ml^-1^ flg22 for 6 h after 5 days of culture. Values are presented as the fold difference from the flg22-untreated wild-type *ACTIN8* gene. Bars indicate SEM from three independent experiments. **(C)** flg22-dependent changes of peroxidase activities. Rosette leaves of 27-day-old seedlings were vacuum infiltrated with or without 0.5 μg ml^-1^ flg22 in the presence of 0.001% Triton X-100. After 24 h incubation, peroxidase activities were measured by using guaiacol as substrate. **(D)** Quantification of stress-inducible lignin of wild-type (WT), *fc1-1*, and *fc2-2* seedlings treated with or without 1 μg ml^-1^ flg22 for 3 days after 3 days of culture.

It is proposed that FC1 is involved in defense response against abiotic and biotic stresses (Singh et al., 2002; Nagai et al., 2007). On the contrary, based on *in silico* co-expression analysis and flg22 (a 22 amino acid peptide of flagellin, which is a bacterial elicitor) dependent oxidative burst assay, Scharfenberg et al. (2015) proposed that FC2 supplies heme not only for photosynthetic cytochromes, but also for proteins involved in stress responses to bacteria. To verify these hypotheses, we determined the effects of flg22 on the expression of *FC1* and *FC2*. As shown in **Figure 8A**, *FC1* was induced by 1 μg/ml flg22 treatment for 6 h in wild type and *fc2-2*, but such induction was not observed in *fc1-1.* Actually, histochemical analysis of *FC1* showed whole plant induction of *FC1* by flg22 treatment (**Figure 8A** inset). The level of induction was approximately fourfold to untreated control, which is less pronounced than other flg22-responsive genes, such as *CYP78, CYP81*, and *MYB41* (**Figure 8B**). It should be noted that consistent with previous observation (Nagai et al., 2007), *HEMA2* was also induced by the flg22 treatment (**Figure 8B**).

To further investigate the function of flg22-induced heme production, we determined the peroxidase activities using guaiacol as a substrate. As shown in **Figure 8C**, peroxidase activities were induced by 0.5 μg/ml flg22 treatment for 24 h in wild-type and *fc2-2*, while such induction was abolished in *fc1-1*, showing that FC1-producing heme is actually involved in the induction of stress-responsive peroxidase activities. However, when we measured the flg22-induced lignin accumulation, the level of lignin accumulation in *fc1-1* was similar to those observed in wild-type and *fc2-2* (**Figure 8D**). Therefore, although the *FC1* induction is abolished, the background heme level in *fc1-1* may be enough for supplying heme to defense-responsive hemoproteins to synthesize stress-responsive lignin under bacterial infections.

## Discussion

In this study, we analyzed the function of ferrochelatase isoforms using T-DNA insertion mutants of *A. thaliana*. Our phenotype analysis is basically consistent with previous studies (Nagai et al., 2007; Scharfenberg et al., 2015; Woodson et al., 2015), although some discrepancies have been found.

### Physiological Importance of FC1-Producing Heme during Development

In the previous studies, except for the stress-responsive induction, the ubiquitous and light-independent expression of *FC1* has been observed under normal conditions tested (Chow et al., 1998; Nagai et al., 2007). Although higher *FC1* expression was detected in roots, effect of its deficiency on root growth has not been observed (**Figure 2A**). Since embryonic lethal phenotype of homozygous *fc1-2* was suggested (Woodson et al., 2011), involvement of FC1-produced heme in embryogenesis has been considered. For *fc1-2*, however, we found some of the homozygous seeds can occasionally germinate (**Figure 1C**). Considering the germinated *fc1-2* stopped their growth at initial seedling stage, it suggests that healthy homozygous *fc1-2* can occasionally form embryo but seedlings are arrested during further development. Our histochemical analysis of *FC1* expression showed prominent staining in primordial tissues of leaves, stipules, and bolting stem in mature seedlings (**Figure 3**). As additional *fc1-1* mutation in *fc2-2* (homozygous *fc1-1 fc2-2* double mutant) caused developmental arrest before or soon after bolting, FC1-produced heme may also be necessary for newly emerging tissues, such as new leaves and bolting stems.

### Involvement of FC2-Producing Heme in Chloroplast Development

On the contrary, FC2-producing heme is mainly supplied for chloroplast development. Consistent with previous studies, *fc2* mutants are abnormally small having pale green rosette leaves with low levels of chlorophylls, carotenoids and several photosynthetic proteins, and their photosynthetic performance was impaired (Scharfenberg et al., 2015; Woodson et al., 2015). Since FC2 deficiency in *fc2-2* caused substantial decrease in total heme contents (**Figure 2D**), it is likely that heme in photosynthetic tissues is predominantly supplied by FC2.

In photosynthetic machineries, FC2-produced heme is mainly incorporated into cytochrome *b_559_* and *b_6_f* complex. In fact, Scharfenberg et al. (2015) reported that cytochrome *b_6_* binding heme was almost undetectable in *fc2-1*, although reduction of cytochrome *f* protein was less pronounced. In our study, the *fc2-2* mutant, which is completely deficient in the *FC2* expression (**Figures 1B** and **4B**), showed a particular decrease in the cytochrome *f* level (**Figure 4B**). Lack of cytochrome *b_6_f* complex could strongly affect the intersystem electron transport process between PSII and PSI. Indeed, *fc2-2* showed lower qP values, which represents enhanced reduction of the plastoquinone pool, than the wild type and *fc1-1* (**Figure 6C**), although *fc2-2* had lower PSII photochemical activity to reduce the plastoquinone pool (**Figure 6B**). The data suggest a strong retardation of electron transport from the plastoquinone pool to downstream components and consistent with the deficiency of the cytochrome *b_6_f* complex in the mutant.

In addition, the *fc2-2* mutation increased relative amounts of LHC antennas to reaction center complexes (**Figure 4B**). Considering the severe reduction of CP43 and CP47 in *fc2-2*, it is likely that the FC2-producing heme is necessary for core antenna complexes of PSII rather than peripheral LHCII antenna complexes. The PAM analyses basically confirmed the results of biochemical analysis of photosynthetic proteins. The analysis revealed that PSII photochemical efficiency represented by *F*_v_/*F*_m_ and F′_v_/F′_m_ was decreased in *fc2-2* (**Figures 6A,B**), in which energetic disconnection between the PSII reaction center and disassembled LHCII complexes may be involved. Consistently, actual photosynthetic efficiency represented by Φ_II_ was considerably decreased in *fc2-2* particularly at middle to high PAR (**Figure 6A**). These changes accompanied increases in thermal dissipation of light energy (Φ_NPQ_) (**Figure 6E**), which occurs in the LHCII antenna (Murchie and Niyogi, 2011), as well as quantum yield of non-regulated energy dissipation (Φ_NO_) (**Figure 6E**). Accumulation of disassembled LHCII increases dissipation of light energy that cannot be used for photochemical reactions, as fluorescence from the antenna system. Moreover, the increase in relative LHCII levels in *fc2-2* may enhance light-induced NPQ activities. Higher NPQ was also observed in the previous study (Scharfenberg et al., 2015). For the reason of higher NPQ, Scharfenberg et al. (2015) suggested the deficiency of PSBO in *fc2-1* may enhance cyclic electron transfer around PSI that induce the abnormally rapid and elevated development of NPQ like the *psbo* mutant (Allahverdiyeva et al., 2005). Although the involvement of the cyclic electron transfer is not clear, our data suggest that relatively larger size of dysfunctional antenna in *fc2-2* may cause higher dissipation of absorbed light energy as heat or fluorescence. Furthermore, deficiency of cytochrome *b_6_f* complexes and consequent impairment of intersystem electron transport may increase non-photochemical energy dissipation with reduced Φ_II_ in *fc2-2.*

For the misbalance between PSII core and the peripheral antenna, the impact of the deficiency of cytochrome *b_559_* in *fc2-2* on photosynthetic activity should be considered (**Figure 4**). It is reported that cytochrome *b_559_* functions in the cyclic electron chain of PSII to protect PSII from photoinhibition (Stewart and Brudvig, 1998). Although the detailed mechanism remains largely unknown about this complex (Plochinger et al., 2016), the deficiency of heme supply in *fc2-2* may affect the function of this complex. Since NPQ involves photoinhibitory component, the possibility that higher NPQ in *fc2-2* is related to cytochrome *b_559_* deficiency cannot be excluded. On the other hand, it is proposed that among the PSII assembly steps, the formation of cytochrome *b_559_*-D2 subcomplex is the initial step that serves as a platform for subsequent incorporation of PSII subunits (Nickelsen and Rengstl, 2013). It is possible that the deficiency of cytochrome *b_559_* in *fc2-2* certainly delayed the PSII assembly of core PSII complex and subsequently resulted in unbalanced accumulation of lower PSII reaction center complexes to LHC antenna.

Alternatively, the possibility that the LHC motif in FC2 is involved in the misbalance between PSII core and the peripheral antenna cannot be excluded. By the presence of the C-terminal LHC motif, FC2 is also categorized as one of eight light-harvesting-like (LIL) proteins in *Arabidopsis* (Jansson, 1999; Engelken et al., 2010). Unlike the LHC, LIL does not appear to be involved in light harvesting, but some of the LIL proteins appear to be at least temporarily associated with the photosynthetic apparatus (Green et al., 1991; Yao et al., 2007).

### FC1-Producing Heme Is Involved in Stress Responses

In this study, our analysis showed induction of *FC1* and repression of *FC2* expression by flg22 treatment and abolishment of *FC1* induction in *fc1-1* (**Figure 8A**). Furthermore, flg22-dependent induction of peroxidase activity was abolished in *fc1-1* (**Figure 8C**). These results are consistent with previous studies (Singh et al., 2002; Nagai et al., 2007). It should be noted that the induction of peroxidase activity was more pronounced in *fc2-2* than wild-type, although similar induction of *FC1* was observed in this mutant (**Figure 8A**). High accumulation of hemoproteins was also observed in the ER-enriched microsomal LM fraction in *fc2-2* (**Figure 7**). It is possible that such a higher heme flux to extraplastidic organelles is caused by higher HEMA1 level in *fc2-2* that increases the global flow of heme biosynthetic pathway since the major FC2-dependent heme production in plastid is prevented in *fc2-2*. Alternatively, a possibility that a decrease in heme supply inside chloroplasts in *fc2-2* may activate HEMA1 activity thorough reducing feedback inhibition of heme cannot be excluded.

On the contrary, Scharfenberg et al. (2015) proposed that heme produced by FC2 is involved specifically in response to biotic stress. Concerning the contradictory conclusions about what isoform produces the stress defensive heme, distinct experimental conditions may cause such discrepancy. For flg22 dependent oxidative burst detection, Scharfenberg et al. (2015) used 4- or 5-week old leaf discs and measured immediate response against flg22 treatment within 21 min. In our previous study (Nagai et al., 2007), stress-induced increase of *FC1* transcripts was observed at least after 15 min of wounding treatment. In this sense, *FC1* must be firstly induced for production of stress defensive heme. Lower heme levels in *fc2-1* may cause higher sensitivity to flg22-dependent oxidative burst (Scharfenberg et al., 2015). Alternatively, the reason why *fc2-1* showed reduced levels of oxidative burst may be related to using aged leaves in the assay. In our histochemical assay of FC1, the expression of FC1 in leaves was substantially lowered in 3-week-old plant when compared to 2-week-old plant (**Figures 3C,D**). Therefore, it is possible that the expression of *FC1* in 5-week-old *fc2-1* leaves is attenuated to lower levels that are not enough for heme production against flg22 dependent oxidative burst. Considering FC2-produced heme is mainly used for chloroplast development while FC1-produced heme is allocated to extraplastidic locations, it is reasonable to assume that the inductive heme production by FC1 under stress conditions is involved in defense mechanism.

## Conclusion

In this study, we showed distinct involvements of FC1 and FC2 in heme supply to subcellular compartments in plant cells. Our data are in accordance with the hypothesis that FC2 produces heme on site for the photosynthetic machinery in the chloroplast and that FC1 is the housekeeping enzyme providing heme cofactor to the entire cell (Nagai et al., 2007; Woodson et al., 2011), but also add new evidence on how heme deficiency in *fc2-2* affects the PSII assembly and the FC1-producing heme is actually involved in the defense mechanism against biotic stresses. In addition, our analysis revealed redundant roles of these isoforms. As null *fc2-2* becomes greener upon development and is fertile, it is apparent that heme produced by minorly expressing *FC1* is partially allocated to plastids and fulfills a sufficient job in the recovery of *fc2-2*. In contrast, as homozygous *fc1-2* could not grow, heme produced by FC2 cannot replace the minor contribution of FC1 on heme production. At present, it is difficult to distinguish whether low but significant accumulation of heme in LM fraction observed in *fc1-1* is supplied by remaining FC1- or FC2-dependent pathway. If it is solely supplied by FC1, it is possible that FC2-produced heme cannot allocate to ER and possibly nucleus for proposed signaling purpose (Woodson et al., 2011).

Besides the housekeeping function, FC1 is assumed to have defense-related function (Singh et al., 2002; Nagai et al., 2007). In addition to the abiotic stresses (Nagai et al., 2007), our study clearly showed that FC1 is also involved in the defense against biotic stresses, such as pathogenesis. As observed in lignin accumulation (**Figure 8D**), so far we are not aware of any increased sensitivities to wounding, pathogenesis and other stresses in *fc1-1*. Since the expression of *FC1* is induced by virus infection (Singh et al., 2002), testing of other biotic stress conditions may give further information about FC1 function.

For the regulation of heme allocation, heme trafficking system is also important. Considering that animal mitochondrial ferrochelatase forms complexes with ABC transporters (Taketani et al., 2003; Chen et al., 2010), distinct binding of each isoform to such transporter protein may occur in plant cell. For heme transfer, heme carrier proteins are also important because of hydrophobic nature of heme. It is demonstrated that heme binding protein (HBP5) interacts with heme oxygenase 1 in plastids probably at the downstream of FC2 in heme catabolic pathway (Hye-Jung et al., 2012), suggesting the existence of specific heme trafficking system. Meanwhile, Vanhee et al. (2011) showed that Golgi-localized TSPO (tryptophan-rich sensory protein) is a heme-binding protein and a potential scavenger of porphyrin via an autophagy-dependent degradation. Further analysis of heme transfer network is necessary in the future.

## Author Contributions

NE carried out main experiments of this manuscript. KK contributed photosynthetic characterization and YS performed flg22-responsive assays. NM contributed genetic analysis of *fc2-2* mutant. KT and RT performed BN PAGE analysis. TM organized and wrote this manuscript.

## Conflict of Interest Statement

The authors declare that the research was conducted in the absence of any commercial or financial relationships that could be construed as a potential conflict of interest.

## Supplementary Material

FIGURE S1 **(A)** Genotyping of *fc1-1 fc2-2* double mutant. Primers are depicted in **Figure 1A**. Since double mutant was infertile, this line is maintained as *fc1-1* homozygous and *fc2-2* heterozygous seeds. **(B)** Photograph of each line. Comparison of heterozygous and homozygous lines of double mutant **(C)**, and *fc2-2* and homozygous line **(D)**. **(E)** Typical phenotype of *fc1-1 fc2-2* homozygous double mutant, which stopped its growth before or soon after bolting.
