# Supplementary material for: Allocation of Heme Is Differentially Regulated by Ferrochelatase Isoforms in Arabidopsis Cells
Source: Front Plant Sci. 2016 Aug 31;7:1326. doi: 10.3389/fpls.2016.01326 (PMC5005420; doi:10.3389/fpls.2016.01326)
Supplement: Supplementary file 1 [file Table_1.DOCX]

**Supplemental Table 1** Oligonucleotides used for PCR and qRT-PCR

| Primer | Sequence | Description |
| --- | --- | --- |
| L | 5’-CTAAGCGTCAATGTGTTATATGTTACAGG-3’ | T-DNA left border |
| F1 | 5’-AGGTATTGGTAATAGACCGGTTCGATTTGG-3’ | *FC1* (Figure 1A) |
| F2 | 5’-GTAGTATCTTGATTTAAGGCTAAAACACAAAG-3’ | *FC2* (Figure 1A) |
| R1 | 5’-AAGACAGAGAATTTCTCTGAGAGCATGACC-3’ | *FC1* (Figure 1A) |
| R2 | 5’-GTCAATACACTCTTCCATCTGC-3’ | *FC1* (Figure 1A) |
| R3 | 5’-TTAAAATAGAAACAAACGTGAGTTTCAAATAAC-3’ | *FC2* (Figure 1A) |
| *ACT8* F | 5’-ACTGTGCCTATCTACGAGGGTTTC-3’ | qRT-PCR (Reference) |
| ACT8 R | 5’- CCCGTTCTGCTGTTGTGGT-3’ | qRT-PCR (Reference) |
| *FC1* F | 5’-ATACCAGAGTCGTGTTGGCCC-3’ | qRT-PCR (*FC1*) |
| *FC1* R | 5’-TCATCGGTGTATGGCTTCAGC-3’ | qRT-PCR (*FC1*) |
| FC2 F | 5’-GAAAATTTGGTTCTCCTAATCAGG-3’ | qRT-PCR (*FC2*) |
| FC2 R | 5’-CATGTGCACTGAAAAATATTAC-3’ | qRT-PCR (*FC2*) |
| *HEMA2* F | 5’-CCTGTGAGATGCGTGAGAA-3’ | qRT-PCR (*HEMA2*) |
| *HEMA2* R | 5’-CTTGACTCCACCGATGCTGA-3’ | qRT-PCR (*HEMA2*) |
| *CYP78* F | 5’-CCACTAATACTTCCCAGATTA-3’ | qRT-PCR (*CYP78*) |
| *CYP78* R | 5’-GATTATCACCTCGGTTCCT-3’ | qRT-PCR (*CYP78*) |
| *CYP81* F | 5’-CTCCAATCTTCTCGTCTATC-3’ | qRT-PCR (*CYP81*) |
| *CYP81* R | 5’-CTCATGCTCAGTATGATGC-3’ | qRT-PCR (*CYP81*) |
| *MYB41* F | 5’-ACAAATGGTCTGCTATAGCT-3’ | qRT-PCR (*MYB41*) |
| *MYB41* R | 5’-CTTGTGTGTAACTGGATCAA-3’ | qRT-PCR (*MYB41*) |
