# Supplementary material for: Allocation of Heme Is Differentially Regulated by Ferrochelatase Isoforms in Arabidopsis Cells
Source: Front Plant Sci. 2016 Aug 31;7:1326. doi: 10.3389/fpls.2016.01326 (PMC5005420; doi:10.3389/fpls.2016.01326)
Supplement: Supplementary file 2 [file Table_2.DOCX]

**Supplemental Table 2** Antibodies used for immunodetection

| **Antibody** | **Description** | **Reference** |
| --- | --- | --- |
| HEMA1 | Rabbit antiserum containing polyclonal antibody against HEMA | Aarti et al. (2007) |
| FC2 | Rabbit antiserum containing polyclonal antibody against FC2. Affinity purified. | Masuda et al. (2003) |
| CYP98A3 | Rabbit antiserum containing polyclonal antibody against CYP98A3. | Nari et al. (2002) |
| PsbA (D1) | Commercially purchased from Agrisera |  |
| PsbD (D2) | Commercially purchased from Agrisera |  |
| PsbC (CP43) | Commercially purchased from Agrisera |  |
| PsbB (CP47) | Commercially purchased from Agrisera |  |
| PsbE (Cyt *b_559_* α) | Commercially purchased from Agrisera |  |
| PetA (Cyt *f*) | Commercially purchased from Agrisera |  |
| LHC | Rabbit antiserum containing polyclonal antibody against LHC. | Tanaka and Tsuji (1982) |
| LHCA1 | Commercially purchased from Agrisera |  |
| LHCA2 | Commercially purchased from Agrisera |  |
| LHCA3 | Commercially purchased from Agrisera |  |
| LHCB1 | Commercially purchased from Agrisera |  |
| LHCB3 | Commercially purchased from Agrisera |  |
| LHCB4 (CP29) | Commercially purchased from Agrisera |  |
| LHCB5 (CP26) | Commercially purchased from Agrisera |  |

Aarti, D., Tanaka, R., Ito, H., and Tanaka, A. (2007). High light inhibits chlorophyll biosynthesis at the level of 5-aminolevulinate synthesis during de-etiolation in cucumber (*Cucumis sativus*) cotyledons. *Photochem Photobiol* 83(1)**,** 171-176. doi: 10.1562/2006-03-06-RA-835.

Masuda, T., Suzuki, T., Shimada, H., Ohta, H., and Takamiya, K. (2003). Subcellular localization of two types of ferrochelatase in cucumber. *Planta* 217(4)**,** 602-609. doi: 10.1007/s00425-003-1019-2.

Nair, R.B., Xia, Q., Kartha, C.J., Kurylo, E., Hirji, R.N., Datla, R., et al. (2002). Arabidopsis CYP98A3 mediating aromatic 3-hydroxylation. Developmental regulation of the gene, and expression in yeast. *Plant Physiol* 130(1)**,** 210-220. doi: 10.1104/pp.008649.

Tanaka, A., and Tsuji, H. (1982). Calcium-induced formation of chlorophyll b and light-harvesting chlorophyll-a/b-protein complex in cucumber cotyledons in the dark. *Biochim. BIophys. Acta* 680(3)**,** 265-270.
