# Supplementary figures and images for: Allocation of Heme Is Differentially Regulated by Ferrochelatase Isoforms in Arabidopsis Cells
Source: Front Plant Sci. 2016 Aug 31;7:1326. doi: 10.3389/fpls.2016.01326 (PMC5005420; doi:10.3389/fpls.2016.01326)

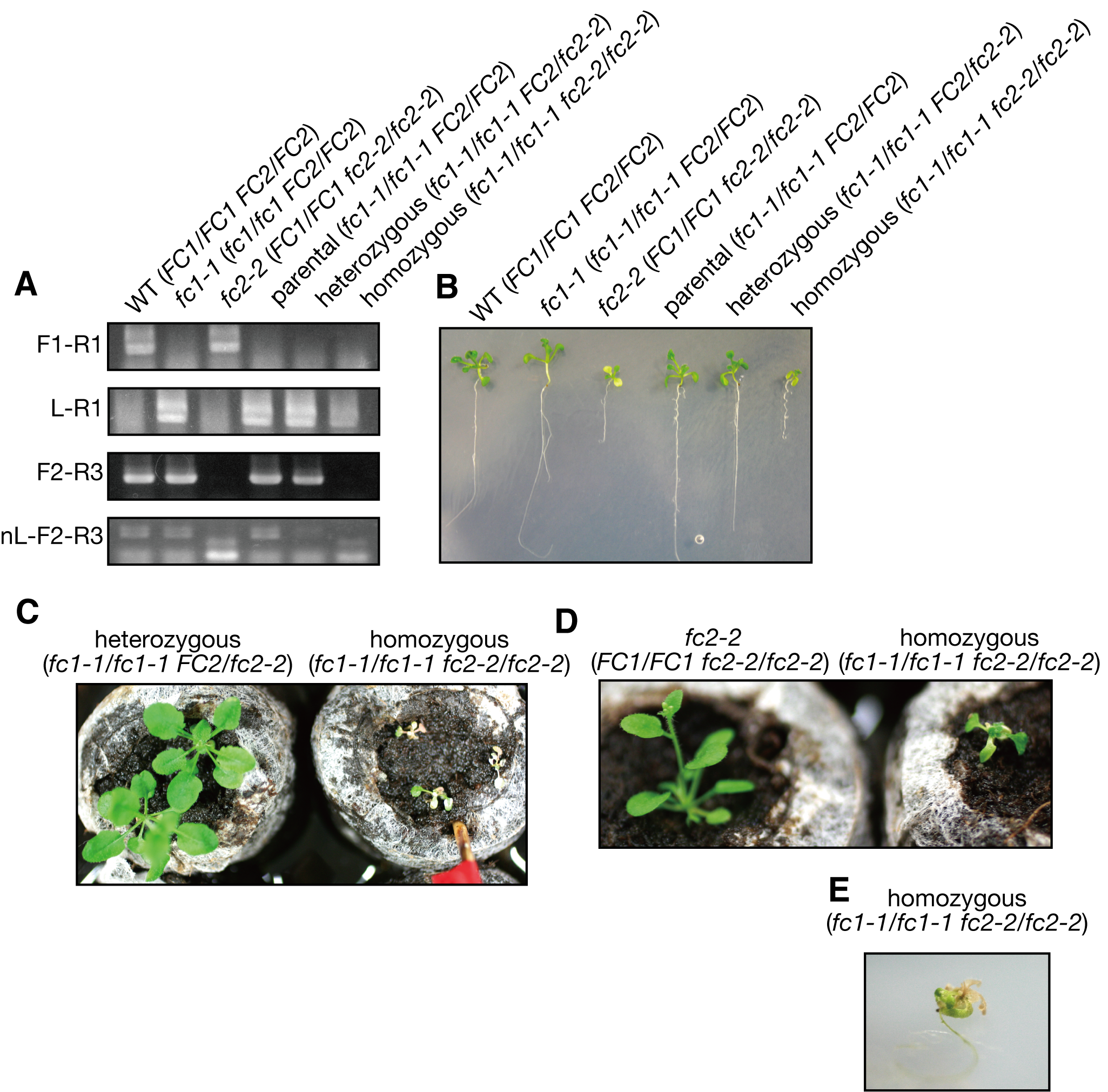

Supplement: FIGURE S1 — (A) Genotyping of fc1-1 fc2-2 double mutant. Primers are depicted in Figure 1A. Since double mutant was infertile, this line is maintained as fc1-1 homozygous and fc2-2 heterozygous seeds. (B) Photograph of each line. Comparison of heterozygous and homozygous lines of double mutant (C), and fc2-2 and homozygous line (D). (E) Typical phenotype of fc1-1 fc2-2 homozygous double mutant, which stopped its growth before or soon after bolting. [file Image_1.TIF]
